# Galvano-Fenton Engineering Solution with Spontaneous Catalyst’s Generation from Waste: Experimental Efficiency, Parametric Analysis and Modeling Interpretation Applied to a Clean Technology for Dyes Degradation in Water

**DOI:** 10.3390/molecules26185640

**Published:** 2021-09-17

**Authors:** Hamza Ferkous, Kaouther Kerboua, Oualid Hamdaoui, Naoufel Haddour, Abdulaziz Alghyamah

**Affiliations:** 1 Laboratory of Environmental Engineering, Process Engineering Department, Faculty of Engineering, Badji Mokhtar—Annaba University, P.O. Box 12, Annaba 23000, Algeria; hamza.ferkous@univ-annaba.org (H.F.); k.kerboua@esti-annaba.dz (K.K.); 2 Department of Second Cycle, Higher School of Industrial Technologies, P.O. Box 218, Annaba 23000, Algeria; 3 Chemical Engineering Department, College of Engineering, King Saud University, P.O. Box 800, Riyadh 11421, Saudi Arabia; aalghyamah@ksu.edu.sa; 4 Laboratoire Ampère, École Centrale de Lyon, 36 Avenue Guy de Collongue, 69134 Écully, France; naoufel.haddour@ec-lyon.fr

**Keywords:** Galvano-Fenton mechanism, degradation kinetics, simulation, parametric analysis, hydroxyl radical, ferrous ion catalyst

## Abstract

In this paper, the degradation of the diazo dye naphthol blue black (NBB) using the Galvano-Fenton process is studied experimentally and numerically. The simulations are carried out based on the anodic, cathodic, and 34 elementary reactions evolving in the electrolyte, in addition to the oxidative attack of NBB by ${\mathrm{HO}}^{•}$ at a constant rate of $3.35\times 10^{7}{\;\mathrm{mol}}^{-1}\cdot m^{3}\cdot s^{-1}$ during the initiation stage of the chain reactions. The selection of the operating conditions including the pH of the electrolyte, the stirring speed, and the electrodes disposition is performed by assessing the kinetics of NBB degradation; these parameters are set to 3, 350 rpm and a parallel disposition with a 3 cm inter-electrode distance, respectively. The kinetics of Fe(III) in the electrolyte were monitored using the principles of Fricke dosimetry and simulated numerically. The model showed more than a 96% correlation with the experimental results in both the blank test and the presence of the dye. The effects of $H_{2}O_{2}$ and NBB concentrations on the degradation of the dye were examined jointly with the evolution of the simulated $H_{2}O_{2}$, ${\mathrm{Fe}}^{2+}$, and ${\mathrm{HO}}^{•}$ concentrations in the electrolyte. The model demonstrated a good correlation with the experimental results in terms of the initial degradation rates, with correlation coefficients exceeding 98%.

## 1. Introduction

Advanced oxidation processes (AOPs) constitute a special class of oxidation techniques [1] that counts those processes based on the generation of reactive oxygen species (ROS) [2] in enough quantity to produce reclaimed effluents. In AOPs, chemical precursors containing oxygen convert to species of high reactivity called ROS. Four major ROS are recognized: superoxide anion radical $O_{2}{}^{•-}$, singlet oxygen ${}^{1}O_{2}$, hydrogen peroxide $H_{2}O_{2}$, and hydroxyl radical ${\mathrm{HO}}^{•}$ [3]. AOPs are then characterized by the capability of exploiting the high reactivity of reactive oxygen species in oxidation for several purposes, such as wastewater treatment [2,4].

Among the ROS, the hydroxyl radical ${\mathrm{HO}}^{•}$ has the highest redox potential after Fluor with a value of 2.8 V/SHE [5] and is non-selective [6]. ${\mathrm{HO}}^{•}$ is capable of attacking organic compounds with constant rates on the order of 10^8^–10^10^ M^−1^·s^−1^ through four pathways: (i) hydrogen abstraction, (ii) combination, (iii) addition of radicals, and (vi) electron transfer [7]. The absolute rate constants are usually determined using the competitive kinetics method between the targeted attacked molecule X and a reference molecule Y for which the absolute kinetic constant of reaction with hydroxyl radical $k_{Y}$ is known. Assuming that the hydroxyl radical will exclusively react with both molecules in the medium, the rate constant $k_{X}$ is determined. The hydroxyl radical oxidative reactions transform the organic molecules into carbon radicals $R^{•}$ or $R^{•}-\mathrm{OH}$, and organic peroxyl radicals ROO in the presence of $O_{2}$. All of these radicals further react with ROS leading to chemical destruction and, in certain cases, total mineralization [2,8,9].

Advanced oxidation processes using hydroxyl radicals as the primary oxidant such as Fenton, photo-Fenton [10], ozonation [11], photocatalysis [12], and sonolysis [13] have emerged as promising new technologies for the degradation of organic pollutants. In recent years, serious consideration has been given to these techniques for the treatment of industrial dye wastes in water [14].

**Figure 1:**
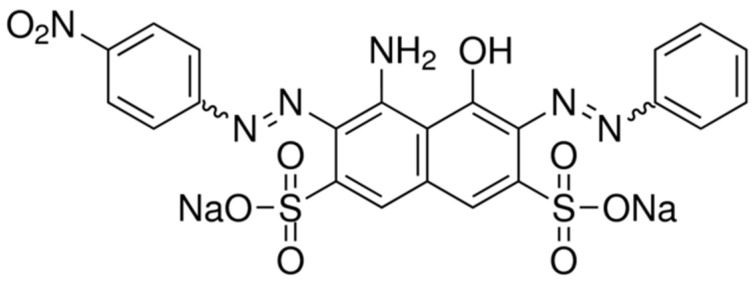
Naphthol blue black molecule.

Among the dyes used in the textile, pulp and paper, plastic, pharmaceutical, food, and paint industries, azoic dyes, characterized by the chromophoric azo bond −N=N−, represent about 70% of annual use [15]. Some azo dyes and their dye precursors have been proven or are suspected to be human carcinogens (as their cleavage can generate toxic aromatic amines) [16], and if this is the case they are then considered emergent pollutants [2]. Naphthol blue black (NBB) is a diazo dye generally used in its pure grade for dyeing foodstuffs, drugs, and cosmetics. NBB is also used as a biological stain and a protein stain in chromatography and electrophoresis. The NBB molecule is presented in Figure 1.

The commercial-grade of NBB is widely employed in the textile industry for dyeing wool, nylon, silk, and textile printing. In addition, it is also used for coloring soaps, anodized aluminum and casein, and preparing writing ink [17]. This dye is potentially present in industrial wastewater and is not degraded by conventional treatment techniques. Therefore, several studies reported the degradation and mineralization of NBB using advanced oxidation processes. For instance, Dalhatou et al. [18] studied the sonochemical degradation of NBB at 278 kHz and investigated experimentally the effects of the acoustic power, dye concentration, and medium matrix on the removal efficiency. Ferkous et al. [19] performed as well an experimental study of the sonolytic degradation of NBB and exhibited the effects of several parameters such as NBB concentration, acoustic intensity, ultrasonic frequency, nature of the dissolved gas, and solution pH. Stylidi et al. [16] examined the mechanistic pathways of the photocatalytic degradation of NBB using solar radiation and aqueous ${\mathrm{TiO}}_{2}$ suspensions. They monitored the adsorption of the dye on the catalyst and its oxidation leading to the formation of intermediates (mainly aromatic and aliphatic acids). More recently, Özcan and Özcan [20] examined the applicability of the electro-Fenton technique to the degradation of NBB, demonstrating that the NBB initially present in a 0.25 mM solution was totally converted to intermediate species within 15 min, while complete decolorization took 180 min. A very high oxidation absolute rate constant of $3.35\;\pm \;0.2\times 10^{10}\;M^{-1}\cdot s^{-1}$ was obtained for the radical attach of hydroxyl radical on NBB. In the work of Özcan and Özcan [20], the absolute rate constant related to the reaction between the hydroxyl radical and the NBB has been determined using the competition kinetics method and benzoic acid as a competitor substance. Since the rate constant is “absolute”, it is intrinsically related to the elementary reaction at the given temperature. Özcan and Özcan’s study [20] was carried out at an external electric current of 60 to 300 mA. In their study, Fenton’s reagent, i.e., $H_{2}O_{2}$ and ${\mathrm{Fe}}^{2+}$, were produced in situ via the cathodic reduction of $O_{2}$ and ${\mathrm{Fe}}^{2+}$. However, the in situ production of ${\mathrm{Fe}}^{2+}$ required the external addition of the iron(III) sulfate pentahydrate in the electrolyte.

Lately, a novel advanced oxidation process patented as ‘Galvano-Fenton’ [21] was studied both experimentally and through simulations. This Galvanic process is based on the spontaneous corrosion of iron without any external current to generate in situ the Fenton catalyst. The study was performed in blank electrolyte [22] as well as in the presence of Acid Orange 7 and phenol as model pollutants [23,24] in order to figure out the pathways of formation and regeneration of the ferrous ion catalyst and the mechanistic aspects that characterize this technique (as compared to classic Fenton and electro-Fenton). Whilst classic Fenton requires stoichiometric yields of added ferrous catalyst in the form of ferrous salt, the Galvano-Fenton process produces the ferrous catalyst in situ by the spontaneous galvanic corrosion of the iron wastes. The chemicals kinetics within the electrolyte is characterized in the classic Fenton process by an initial yield of ferrous catalyst, whose regeneration is relatively slow, which slow-down the reaction rates as soon as a considerable number of ferrous ions is transformed into ferric ions. In contrast, the ferrous catalyst is produced continuously in the Galvano-Fenton process, which compensates the slow regeneration of ferrous ions from ferric ions formed during the catalytic reaction of decomposition of hydrogen peroxide. Moreover, the electro-Fenton process relies on the electrochemical reactions evolving at the electrodes under the effect of external electric current, which is equivalent to energy consumption. On the other hand, the Galvano-Fenton process is governed by spontaneous corrosion within a galvanic cell, wherein the iron waste plays the role of the sacrificial anode. Thus, the Galvano-Fenton process does not require any external current and, on the contrary, produces energy along with the oxidation of target pollutants. From a technological point of view, the energetic outcomes of the Galvano-Fenton process have been recently studied as a novel approach for energy from waste [25], and estimations have been made in terms of energy production and saving, using the basic configuration as well as an upgraded one relying on a pre-immersion stage of the electrodes in the electrolyte [24]. Economically speaking, a direct comparison with the classic Fenton process clearly shows that the Galvano-Fenton eliminates the need for ferrous salt addition, which prevents at least an additional operation cost related to the use of ferrous sulfate heptahydrate salt as a catalyst source [26]. The Galvano-Fenton process runs without any external current supply, which eliminates any operation cost related to the energy consumption of the process itself, which is a supplementary cost to consider with the electro-Fenton process [27,28].

In the present paper, the main objective of the study is to assess the efficiency of the Galvano-Fenton process in the degradation of naphthol blue black and interpret the observed effects based on the simultaneous electrochemical and chemical kinetics simulated numerically. The present paper is rather focused on the performance of the GF technique in terms of the degradation efficiency of the dye. This investigation is carried out experimentally, and modeled, simulated, and interpreted numerically. It covers a parametric analysis related to the design parameters, namely the stirring speed, the electrodes disposition, and the electrolyte pH as well as the effects of the dye and hydrogen peroxide concentrations.

## 2. Results and Discussion

### 2.1. Selection of Operating Conditions

In order to perform the Galvano-Fenton degradation of NBB, the experimental setup has been optimized in terms of operating conditions comprising the disposition of the electrodes, the stirring speed, and the pH of the electrolyte solution through a preliminary study.

**Figure 2:**
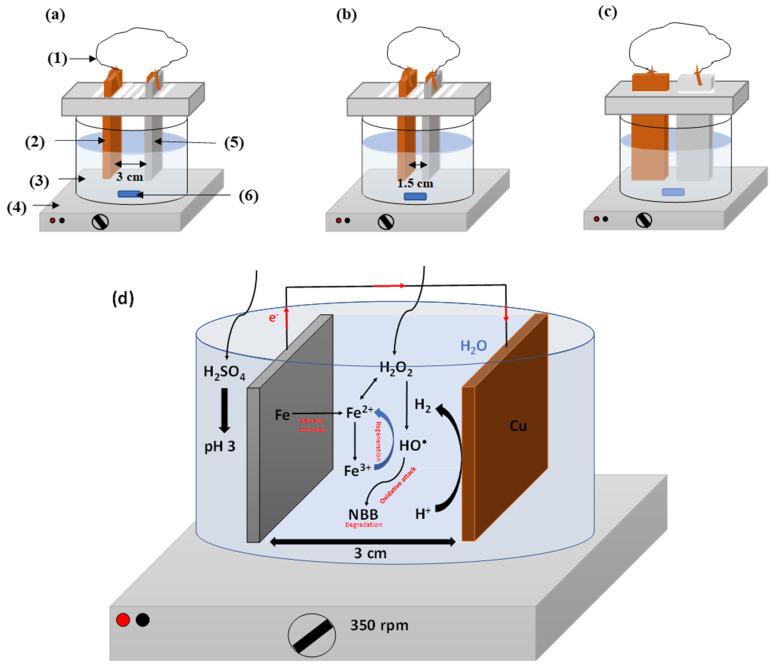
Schematic representation of the three dispositions of electrodes in the Galvano-Fenton process: parallel with 3 cm inter-electrodes distance: (1) external electric wire; (2) copper plate sheet; (3) electrolyte; (4) magnetic stirrer; (5) iron plate sheet; (6) magnetic bar (**a**), parallel with 1.5 cm inter-electrodes distance (**b**) and aligned electrodes with profile disposition (**c**), and the final adopted experiment (**d**).

**Figure 3:**
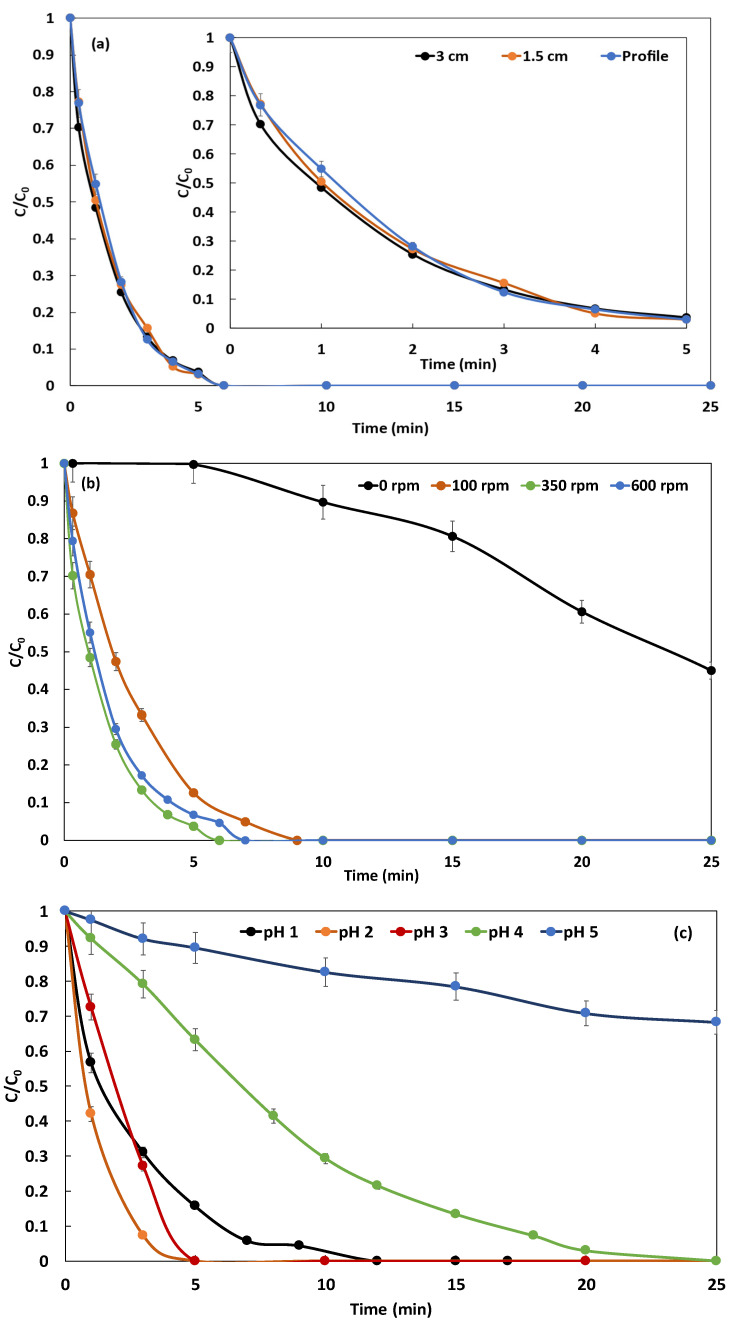
Effect of the disposition of the electrodes (**a**), the stirring speed (with a zoom on the first 5 min) (**b**), and the electrolyte pH (**c**) on the kinetics of degradation of NBB (NBB concentration: 5 mg·L^−1^, 162 µM of added H_2_O_2_, pH 3 when not varied, stirring speed: 350 rpm when not varied).

Three different dispositions were tested, namely (i) parallel iron and copper plate sheets distant of 3 cm, (ii) parallel electrodes distant of 1.5 cm, and (iii) aligned electrodes in profile disposition as represented in Figure 2. The kinetics of degradation of NBB is shown in Figure 3a for the three dispositions of electrodes. Figure 3a demonstrates that the three curves are superimposed which proves that the disposition does not affect the ions transfer within the electrolyte. This observation can be explained by the stirring speed of 350 rpm ensuring perfect mixing and contact solution-electrode, regardless of the position and distance. Consequently, and for maneuverability purposes, we opt for a parallel disposition of electrodes and inter-electrodes distance of 3 cm.

In order to optimize the stirring speed, the Galvano-Fenton degradation of NBB is monitored vs. time: (i) in the absence of stirring, and at (ii) 100, (iii) 350, and (vi) 600 rpm. The results are reported in Figure 3b. In the absence of stirring, it is observed that the kinetics of degradation of NBB becomes very slow and barely attains 50% within 25 min. In this case, the mechanism of the Galvano-Fenton process is clearly limited by the mass transfer of ions in the electrolyte. At 100 rpm, the kinetics of degradation is significantly accelerated and the dye is totally degraded within 9 min. The mechanism of degradation starts directly when the electrodes are immersed and hydrogen peroxide is added, no latency is observed. Increasing the stirring speed to 350 rpm induces an improvement of the total degradation time, which passes to 5 to 6 min. However, this trend is not indefinitely valid, when the stirring speed is further increased to 600 rpm, the total degradation time becomes slightly longer and reaches 7 min. This trend reversal due to the augmentation of stirring speed was previously observed by Bayar et al. [29] and could be explained by the highly turbulent flow created by fast stirring and leading to the disturbance of ions transfer from and towards the electrodes. Hence, 350 rpm is the optimal stirring speed and will be adopted in the following experimental setups.

The effect of the pH is investigated by carrying out the degradation of 5 mg·L^−1^ of NBB while adjusting the acidity of the electrolyte to pH 1, 2, 3, 4, and 5. The kinetics of degradation in the five cases are reported in Figure 3c. It is observed that at pH 5, the degradation becomes very slow and only 35% of the dye is degraded within 25 min. At pH 4, the degradation is significantly accelerated, and the dye is totally degraded within the experiment duration. However, the fastest degradation is observed at pH 3 and pH 2, and total degradation is achieved within 6 and 5 min, respectively. When the pH is decreased to 1, the reverse effect is noticed and the complete degradation is attained within 12 min. Chang et al. [30] studied the effect of pH varying in the range of 2 to 5 on Fenton reaction and demonstrated using salicylic acid as a trapping reagent that hydroxyl radical has the highest formation rate constant at pH 3.0 and pH 4.0. Burbano et al. [31] also investigated the influence of pH on the degradation of MTBE with Fenton reagent, demonstrating that the highest reduced fraction of MTBE is observed at pH 3 among the range from 3 to 7. The authors explained their observation by the fact that ${\mathrm{HO}}^{•}$ radicals are expected to exhibit the highest oxidative power at this pH. Safarzadeh-Amiri et al. [32] carried out a comprehensive study linking the pH value to iron speciation in the Fenton reaction. They demonstrated that the $\mathrm{Fe}\left(\mathrm{II}\right)/\mathrm{Fe}\left(\mathrm{III}\right)-H_{2}O_{2}$ system has its maximum catalytic activity at a pH of about 2.8–3.0. At higher pH, the ferric ion precipitates as ferric hydroxide [33], and at lower pH, the complexation of Fe(III) with $H_{2}O_{2}$ is inhibited. For the aforementioned reasons, and owing to the slight difference observed in NBB degradation at pH 2 and 3, the value of pH 3 is adopted for the following experiment in order to reduce the consumption of acid and facilitate the neutralization of the electrolyte solution once the dye is degraded.

### 2.2. Monitoring of Fe(III) and $HO^{•}$ Kinetics

**Figure 4:**
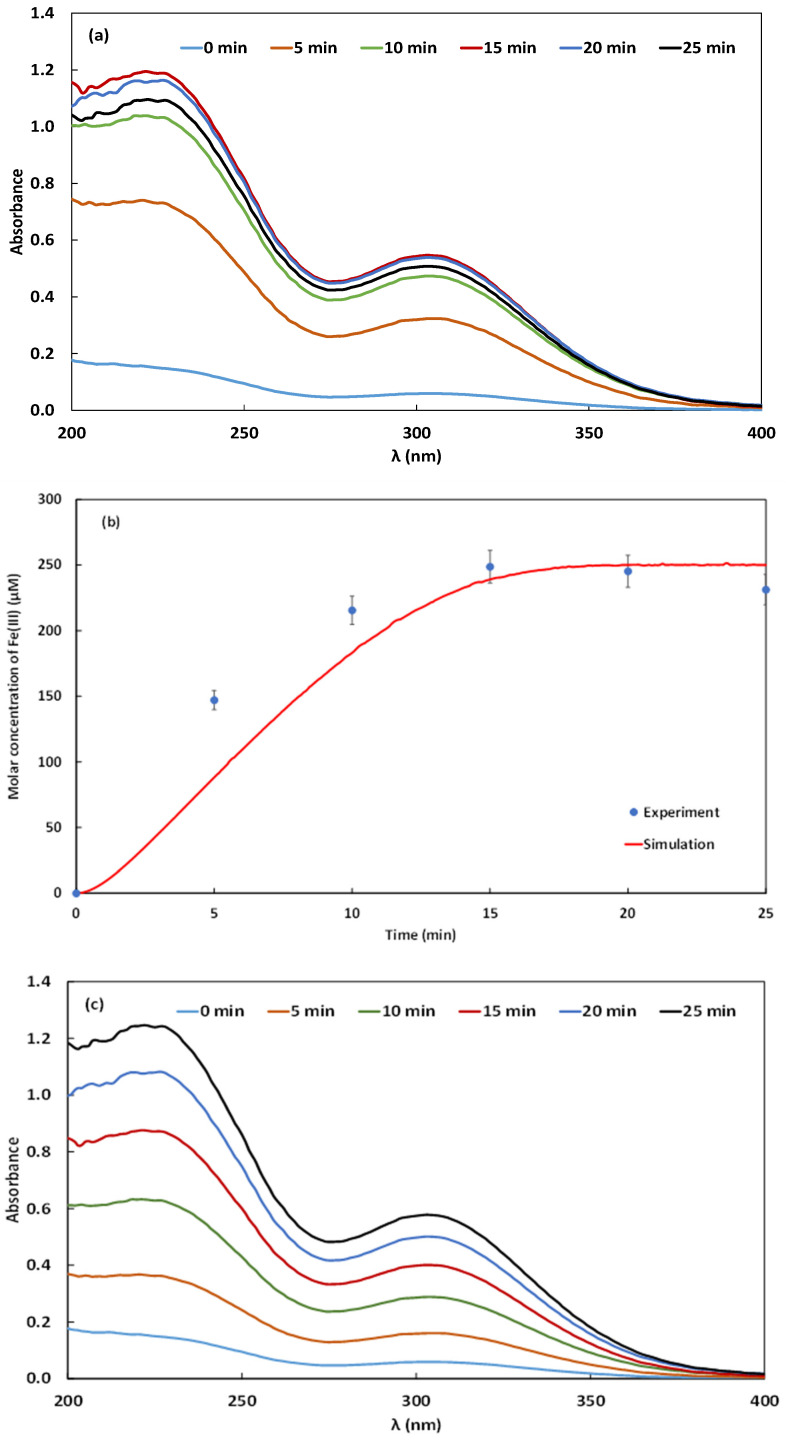

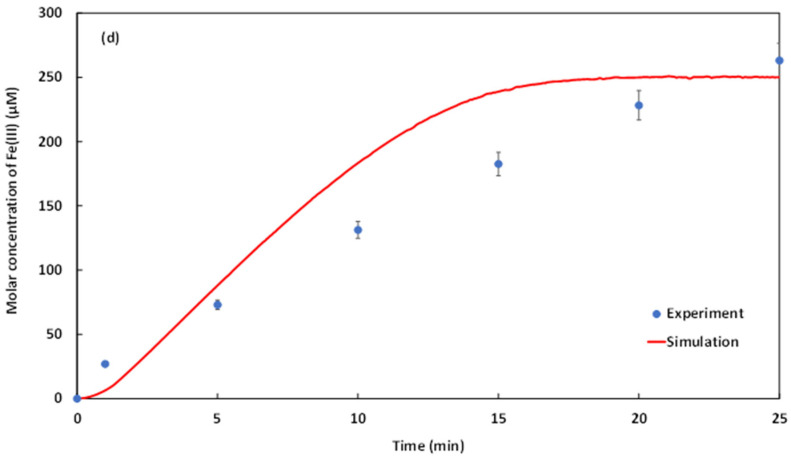
UV-vis spectra describing the kinetics of Fe(III) in the electrolyte obtained in the absence of the dye (**a**) and its corresponding comparison of experimental and simulated results (**b**), and the presence of the dye at an initial concentration of 5 mg·L^−1^ (**c**) with its corresponding comparison of experimental and simulated results (**d**) (pH 3 and 162 µM of added H_2_O_2_).

The ferric species emerging in the electrolyte during the Galvano-Fenton process were quantified based on the Fricke dosimetry and reduced to ferric ions whose absorbance is measured using a spectrophotometer. The kinetics of iron at its +3 oxidation state is monitored experimentally in the absence of the dye, i.e., blank test, and in the presence of the dye at 5 mg·L^−1^ and their related spectrophotometric spectra are presented as a function of time in Figure 4a,c, respectively, and the resulting kinetics are reported in Figure 4b,d, respectively, with the corresponding simulated evolution of Fe(III) yields vs. time. To perform the numerical simulations, the current density has been identified using an iterative method with a step of 0.00001 A·cm^−2^. The best-fitting curves have been obtained with a current density of 0.0012 A·cm^−2^. In Figure 4b, experimental and simulated results demonstrate a correlation coefficient of 96.24%, Fe(III) concentration exhibits a final value attained at t=25 min of 230 to 250 µM, in the absence of NBB.

**Figure 5:**
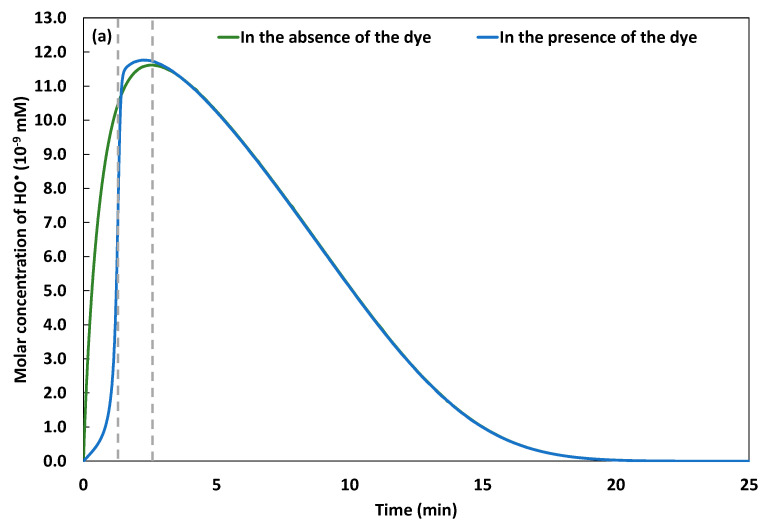

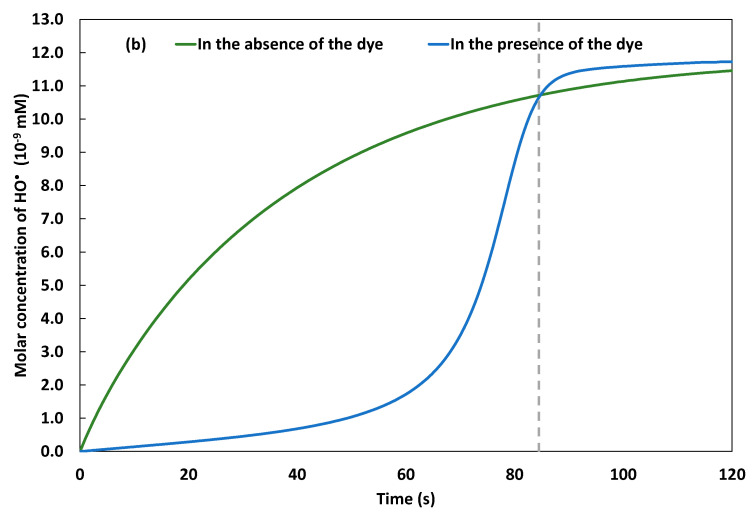
Simulated production of hydroxyl radical in the absence and in the presence of the dye (pH 3, initial concentration of the dye when present 5 mg·L^−1^ and 162 µM of added H_2_O_2_) (**a**). Zoom on the first 120 s (**b**). The dotted lines point out *t* = 86 s and *t* = 155 s.

In the presence of NBB, Figure 4d shows a correlation coefficient of 96.62% between the experimental and numerical results, which validates the model results. In this case, the concentration of Fe(III) attains by the end of the experimental duration 250 to 263 µM. The monotonous increase of Fe(III) concentration in the electrolyte, slightly decelerated when approaching t=25 min, reveals the slow regeneration of ferrous ions within the experiment’s duration, demonstrated previously by Gasmi et al. [22]. Simultaneously, the continuous release of ${\mathrm{Fe}}^{2+}$ by the galvanic corrosion of iron plate sheet, and the regenerated ${\mathrm{Fe}}^{2+}$ through the reaction (2)–(5), (10), (12), and (30)–(34) reported in Table 1 catalyze the decomposition of $H_{2}O_{2}$ through reaction (1) and generates ${\mathrm{HO}}^{•}$. The production of ${\mathrm{HO}}^{•}$, governed by Equations (4) and (8), is simulated in the absence and in the presence of NBB by resolving the system of non-linear differential equations presented in the “Numerical modeling and simulation” section. The obtained results are presented in Figure 5a,b.

Figure 5a shows a gradual increase of the concentration of hydroxyl radical in the electrolyte, until attaining the maximum value of $1.17\times 10^{-8}\;\mathrm{mM}$ at t=155 s, in the absence of the dye. However, in the presence of the dye, the curve of ${\mathrm{HO}}^{•}$ concentration vs. time exhibits a clear gap between $t_{0}$ and t=86 s, as shown in Figure 5b. During this timeslot, ${\mathrm{HO}}^{•}$ is drastically consumed by the NBB molecules, owing to its high concentration. The generation of the hydroxyl radical through the reactions 1, 24, and 25, reported in Table 1, and its consumption through the reactions 5, 13, 16, 17, 22, and 23, presented in Table 1, and the initiation reaction, reported in Table 2, occur simultaneously with comparable rate constants. This results in added positive and negative reaction rates applied to hydroxyl radical, its yield knows competitive opposite kinetics. The competition leads to the apparition of a maximum concentration of $1.17\times 10^{-8}\;\mathrm{mM}$ at t=120 s. The degradation of the dye, particularly during the initiation stage, is examined in the following section.

### 2.3. Effect of H_2_O_2_ Concentration

**Figure 6:**
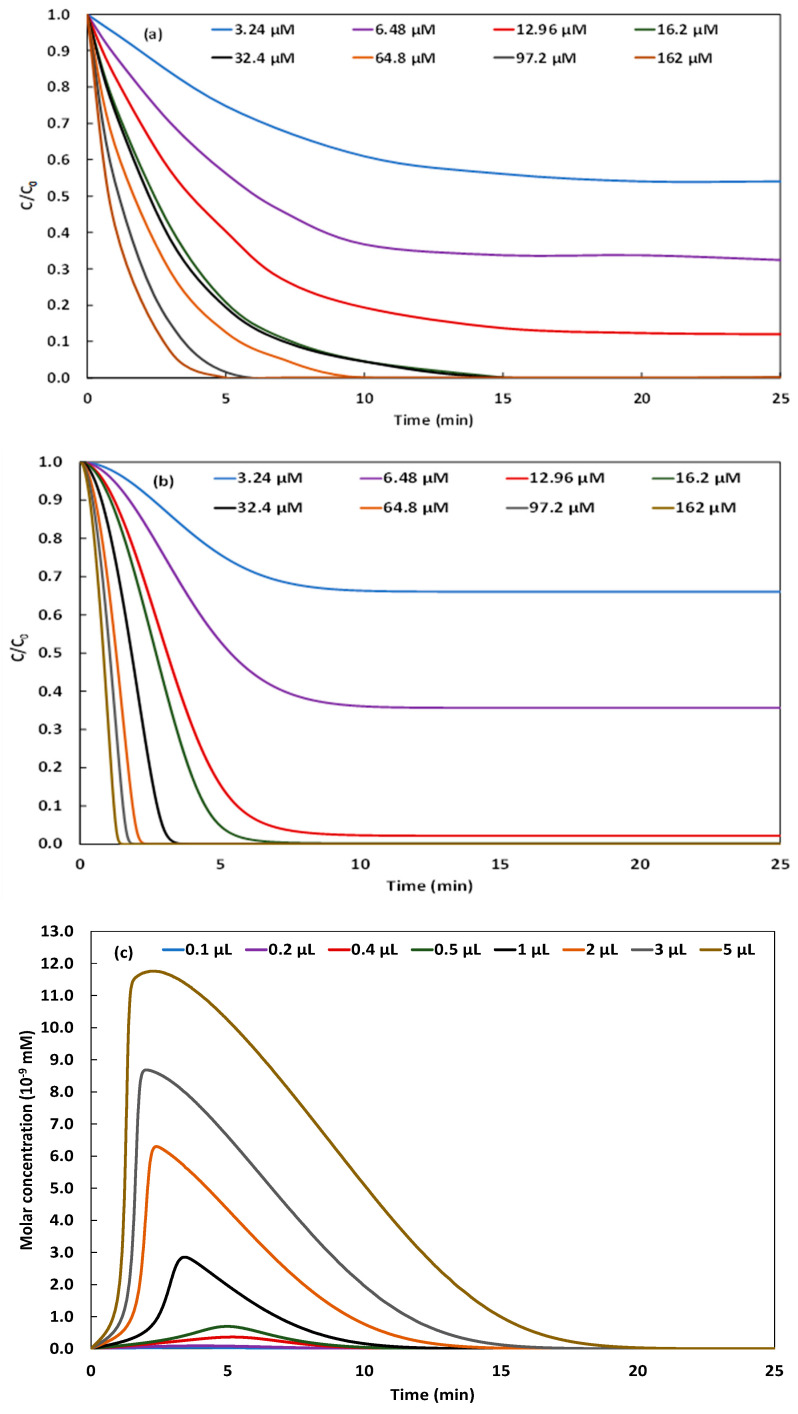

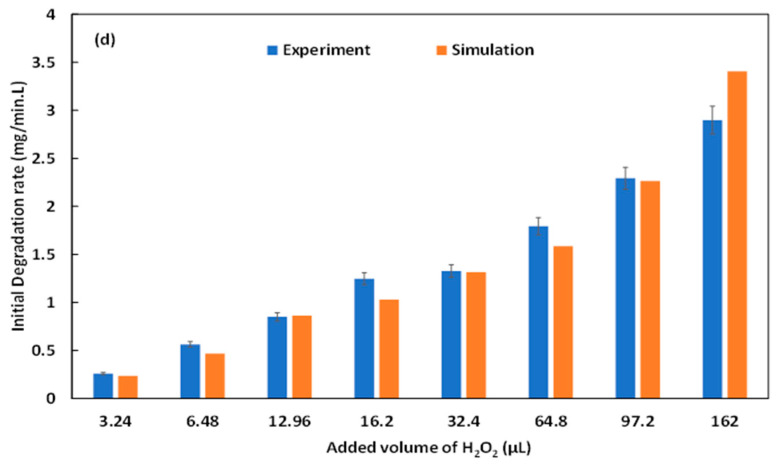
Experimental (**a**) and simulated (**b**) kinetics of Galvano-Fenton degradation of NBB vs. time, and their corresponding simulated kinetics of hydroxyl radical production (**c**), and comparison of the initial degradation rates obtained experimentally and numerically (**d**) with different concentrations of added hydrogen peroxide (pH 3 and initial concentration of NBB: 5 mg·L^−1^).

**Figure 7:**
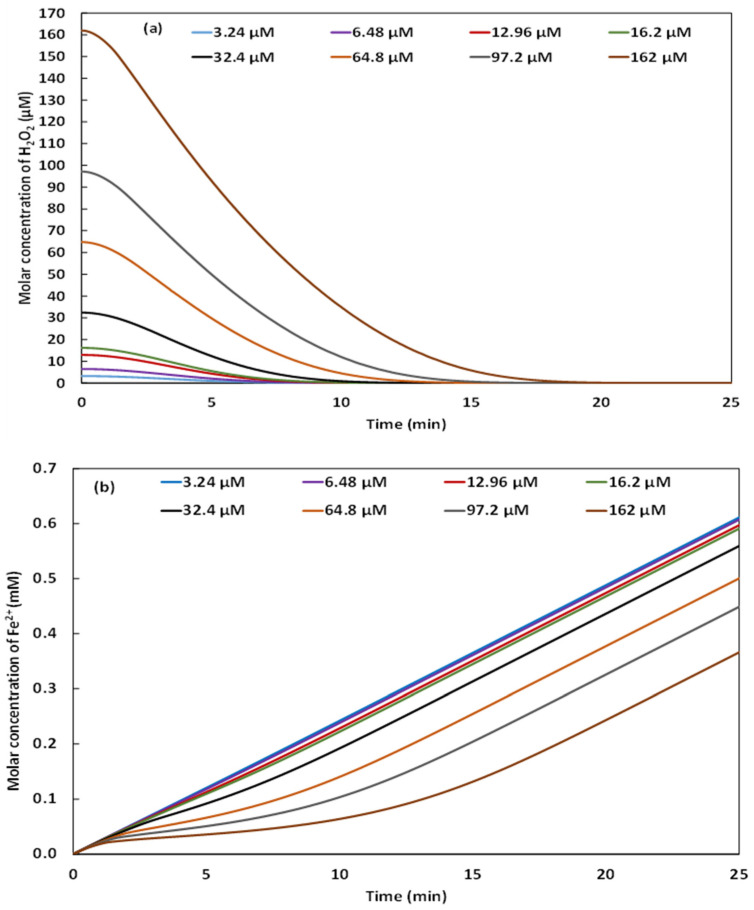
Simulated kinetics of $H_{2}O_{2}$ consumption (**a**) and ${\mathrm{Fe}}^{2+}$ generation (**b**) in the electrolyte with different concentrations of added hydrogen peroxide (pH 3 and initial concentration of NBB: 5 mg·L^−1^).

$H_{2}O_{2}$ is the oxidizing species of the Fenton reagent ($H_{2}O_{2}/{\mathrm{Fe}}^{2+})$. In the present study of the Galvano-Fenton process, the effect of $H_{2}O_{2}$ concentration is investigated by monitoring and simulating the degradation of 5 mg·L^−1^ of NBB considering the addition of $H_{2}O_{2}$ at concentrations of 3.24, 6.48, 12.96, 16.2, 32.4, 64.8, 97.2, and 162 µM in the electrolyte. Figure 6a,b present, respectively, the kinetics of degradation of NBB obtained experimentally and numerically. The simulation accounts for the initiation stage of the chain reactions mechanism of degradation of NBB, as suggested by Özcan and Özcan [20]. Hence, the experimental and simulated kinetics are particularly compared at the initial stage, special attention is paid to the initial degradation rates believed to correspond to the hydroxyl attack on NBB molecules. Both Figure 6a,b demonstrate that the higher the concentration of $H_{2}O_{2}$ in the electrolyte, the faster the degradation of the dye. For initial concentrations of 16.2, 32.4, 64.8, 97.2, and 162 µM, the total degradation is observed within the study timeframe at 15, 15, 10, 6, and 5 min, respectively. The analysis of the simulated evolutions of $H_{2}O_{2}$ yields, reported in Figure 7a, demonstrates a total consumption of the added yields within the study timeframe. However, at concentrations of 3.24, 6.48, and 12.96 µM, $H_{2}O_{2}$ is totally consumed in almost 10 min, the oxidation of the dye is then limited by the hydrogen peroxide which is totally consumed before generating the necessary hydroxyl radical to degrade all of the molecules of NBB. The simulated evolution of the concentration of ${\mathrm{HO}}^{•}$ is reported in Figure 6c.

Generally, ${\mathrm{HO}}^{•}$ yield increases rapidly from the beginning of the Galvano-Fenton process until attaining a maximum value within the first 5 min then decreasing. Notably, the higher the concentration of $H_{2}O_{2}$, the higher the concentration of generated ${\mathrm{HO}}^{•}$ and the earlier the maximum value occurs. For instance, with an initial concentration of hydrogen peroxide of 3.24 µM, the concentration of hydroxyl radical reaches $3.73\times 10^{-11}\;\mathrm{mM}$ at 3.5 min, while with 162 µM concentration of $H_{2}O_{2}$, ${\mathrm{HO}}^{•}$ attains $1.17\times 10^{-8}\;\mathrm{mM}$ at 2 min. This maximum concentration of hydroxyl radicals feeds the initiation stage of the chain reactions mechanism of the degradation of NBB. The ferrous ions are released continuously in the electrolyte, once the hydrogen peroxide is totally consumed, the evolution of ${\mathrm{Fe}}^{2+}$ concentration shows a quasi-linear trend as demonstrated in Figure 7b, owing to the braking of reaction (1) reported in Table 1. The initial rates of degradation, calculated from experimental and numerical results based on the linearly decreasing evolution shown in Figure 6a,b during the first stage of degradation, are reported in Figure 6d. The comparison of experimental and simulated initial rates of degradation of NBB exhibits a correlation coefficient of 98.33%, which proves that the kinetic constant determined by Özcan and Özcan [20] and adopted in the present work describes well the initiation reaction of the oxidative attack of NBB by hydroxyl radical, reported in Equation (8). The highest initial rate of degradation is recorded with an initial concentration of $H_{2}O_{2}$ of 162 µM, its value is in the order of 2.9 to 3.4 mg·L^−1^·min^−1^ of NBB. The lowest value is attained with an initial concentration of $H_{2}O_{2}$ of 3.24 µM, and equals 0.23 to 0.26 mg·L^−1^·min^−1^ of NBB. The initial degradation rate increases monotonously between the lowest and the highest values with the increase of the volume of $H_{2}O_{2}$ added at $t_{0}$.

### 2.4. Effect of NBB Initial Concentration

**Figure 8:**
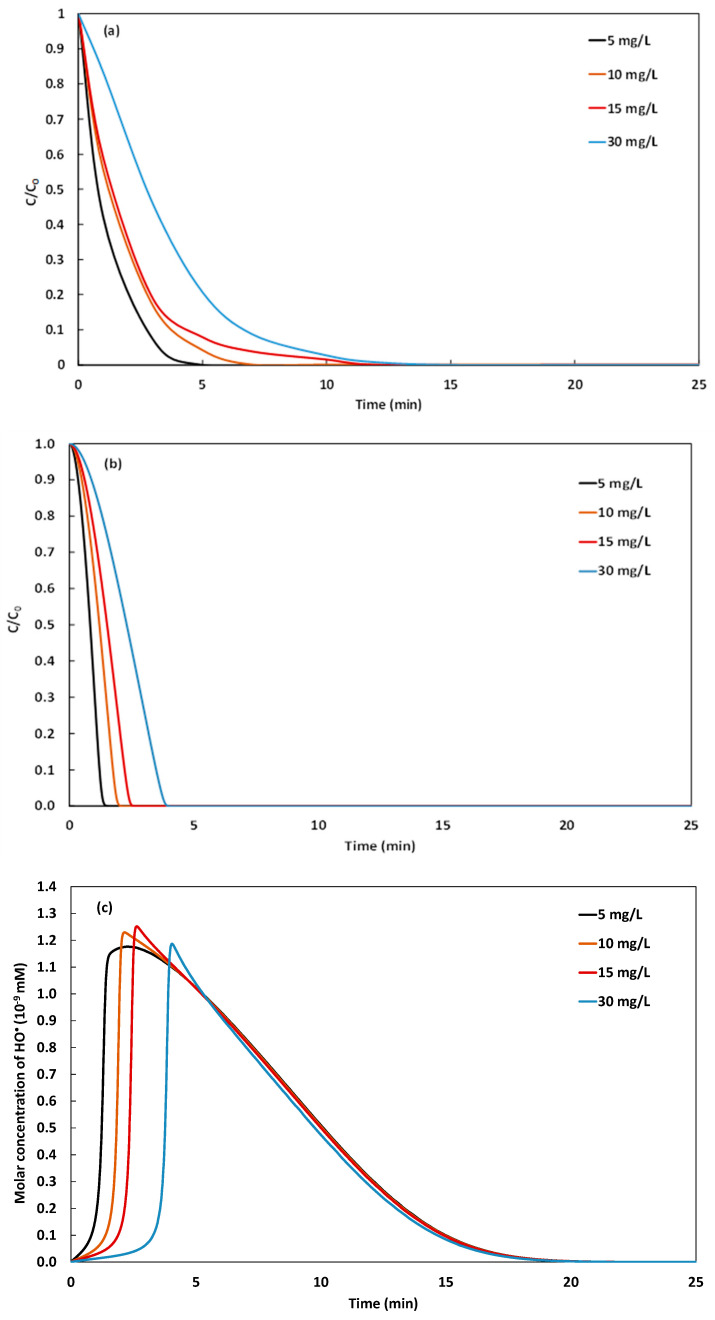

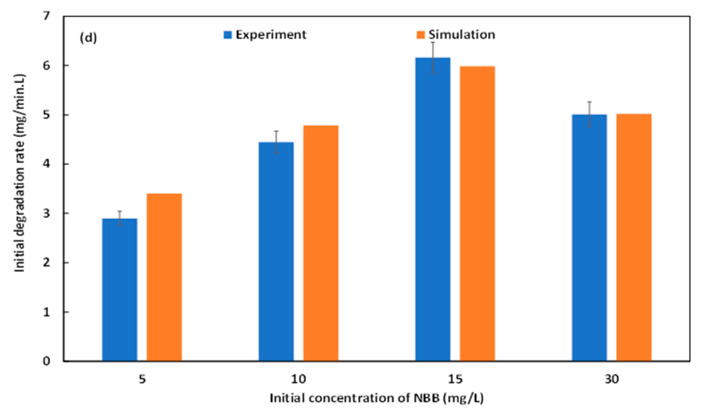
Experimental (**a**) and simulated (**b**) kinetics of GF degradation of NBB vs. time, their corresponding simulated kinetics of hydroxyl radical production (**c**), and comparison of the initial degradation rates obtained both experimentally and numerically (**d**) with different initial concentrations of NBB (pH 3 and 162 µM of added H_2_O_2_).

The effect of the initial concentration of the dye is investigated over the range of 5, 10, 15, and 30 mg·L^−1^, and the kinetics of degradation are monitored and reported in Figure 8a. The simulations performed using the same values on initial concentrations of the dye are reported in Figure 8b and their corresponding kinetics of hydroxyl radical are presented in Figure 8c. From Figure 8a,b, it is observed that the increase of the concentration of the dye induces the extension of the necessary time for total degradation, which passes for instance from 5 min with 5 mg·L^−1^ of NBB to 12 min with 30 mg·L^−1^ of NBB. However, the initial concentration of the dye influences the initial degradation rate, it is observed in Figure 8c that it increases from the order of 3 mg·L^−1^·min^−1^ with 5 mg·L^−1^ of NBB to almost 6 mg L^−1^·min^−1^ with 15 mg·L^−1^ of NBB. However, it decreases again when augmenting the initial concentration of the dye to 30 mg·L^−1^, the initial degradation rate drops to the order of 5 mg·L^−1^·min^−1^. The experimental and the numerical results are correlated to 99.64%. The analysis of the simulated kinetics of hydroxyl radical, presented in Figure 8c, shows the same evolution trend depicted previously versus time. The concentration of ${\mathrm{HO}}^{•}$ increases rapidly until attaining a maximum by the beginning of the Galvano-Fenton degradation of NBB, then decreases again to very low values. We pay particular attention to the maximum concentrations of ${\mathrm{HO}}^{•}$ varying as a function of the initial concentration of the dye. With 5 mg·L^−1^ of NBB, the highest concentration of hydroxyl radical is attained at 120 s and equals $1.17\times 10^{-8}\;\mathrm{mM}$, when increasing the initial concentration of the dye to 10 then 15 mg·L^−1^, the maximum concentrations of ${\mathrm{HO}}^{•}$ is slightly increased to $1.23\times 10^{-8}$ and $1.25\times 10^{-8}\;\mathrm{mM}$ at respective instants of 135 s and 163 s, respectively. Finally, when the concentration of NBB is increased to 30 mg·L^−1^, the highest concentration of ${\mathrm{HO}}^{•}$ occurs at 253 s and equals $1.16\times 10^{-8}\;\mathrm{mM}$, which explains the observed decrease in the initial degradation rate. The diminution of the concentration of hydroxyl radical can be explained by the simultaneous rapid consumptions of the Fenton reagent and the generated hydroxyl radical. Hence, the yield of ${\mathrm{HO}}^{•}$ contributing to the oxidative attack of NBB molecules is not compensated by the decomposition of $H_{2}O_{2}$. In this case, a higher volume of added hydrogen peroxide is necessary to maintain the initial degradation rate proportional to the concentration of the dye.

## 3. Materials and Methods

### 3.1. Experimental Setup

A glass beaker of 0.4 L capacity is used as a batch reactor and filled with 300 mL of distilled water. For experiments realized in the presence of the dye, appropriate yields of naphthol blue black powder (Abbreviation: NBB; Acid Black 1; C.I. number: 1064-48-8; chemical class: azo dye; molecular formula: C_22_H_14_N_6_Na_2_O_9_S_2_, molecular weight: 616.49 g·mol^−1^, supplied by Sigma-Aldrich) were added to distilled water to reach the intended concentration (5, 10, 15 or 30 mg·L^−1^, according to the experiment). The pH of the solution is adjusted to 3 by adding sulfuric acid (0.1 N, supplied by Sigma-Aldrich). At $t_{0}=0$, iron $\left(E^{0}({\mathrm{Fe}}^{2+}/\mathrm{Fe})=-0.44\;V\;\mathrm{vs}.\;\mathrm{SHE}\right)$ and copper $\left(E^{0}({\mathrm{Cu}}^{2+}/\mathrm{Cu}\right)=0.34\;V\;\mathrm{vs}.\;\mathrm{SHE})$ plate sheets, put in contact using an external electric wire, are both immersed in the solution at a 3 cm distance, offering 20 cm^2^ of electrode-solution contact surface each. Simultaneously, a specific concentration of $H_{2}O_{2}$ (3.24, 6.48, 12.96, 16.2, 32.4, 64.8, 97.2, and 162 µM in the electrolyte, according to the experiment) is added from a stock solution ($30\%\;w,\;d=1.11\;\mathrm{kg}\cdot L^{-1}$, $C_{0}=9.795\;M$ purchased from Sigma-Aldrich). A magnetic stirrer placed at the bottom ensures mixing of reactants towards/from the electrodes at 100, 350, and 500 rpm, and enhance consequently mass transfer. Once immersed in the batch reactor containing the electrolyte, both connected metals form a galvanic cell and induce galvanic corrosion of iron which plays the role of the sacrificial anode and continuously releases ferrous ions catalyst into the solution. The electrons resulting from the oxidation of metal iron pass to the cathode through the external wire circuit, which simultaneously produces electrical energy. At the cathode, electrons allow the reduction of protons forming hydrogen gas. All the experiments were carried at an ambient temperature of 25 °C and over 25 min, judged sufficient to monitor the intended kinetics resulting from the covered operating conditions.

Samples are taken periodically from the electrolyte solution to assess the concentrations of NBB and Fe(III). The concentration of NBB is determined by measuring the absorption at 620 nm using a UV–visible spectrophotometer (Lightwave II). The kinetics of Fe(III) is monitored in the electrolyte using the principle of Fricke dosimetry [34]. Thus, the concentration of Fe(III) is estimated through spectrophotometric absorption at 303 nm $\left(\varepsilon =2197{\;L\cdot \mathrm{mol}}^{-1}\cdot {\mathrm{cm}}^{-1}\right)$.

### 3.2. Numerical Modeling and Simulation

The numerical model proposed in the present work intends to describe the simultaneous kinetics of the galvanic and electrolytic reactions in order to explain the experimental observations established in blank tests and related to the degradation of NBB. The simulations intend as well to figure out the action mechanism of the parameters investigated in the parametric analysis, particularly in terms of the generation of hydroxyl radicals. The kinetics of the galvanic corrosion of the iron metal is described by Faraday’s law [35].

(1) $$\frac{d[X_{k}]}{dt}=\pm \frac{i_{corr}}{nFV}$$

This equation is applied to the species $X_{k}$ involved in the electrochemical reactions; namely Fe, ${\mathrm{Fe}}^{2+}$, $H^{+}$, and $H_{2}$. The anodic and cathodic current animate reactions (I) and (II), respectively, which are reported in Table 1 [22]. Reaction (I) describes the generation of ferrous ions in the electrolyte, and once $H_{2}O_{2}$ added, a rapid decomposition of the latter starts, catalyzed by ${\mathrm{Fe}}^{2+}$, a chain mechanism then evolves in the electrolyte, as described by the reactions (1) to (34) reported in Table 1. Considering the generation of radical and ionic species following the decomposition of $H_{2}O_{2}$ in the presence of ${\mathrm{Fe}}^{2+}$, the formation of intermediate ferrous salt ${\mathrm{FeSO}}_{4}$ and ferric complexes ${\mathrm{FeOH}}^{2+}$, $\mathrm{Fe}\left(\mathrm{OH}\right){\mathrm{HO}}_{2}{}^{+}$ and $\mathrm{Fe}{\left({\mathrm{HO}}_{2}\right)}^{2+}$, and the reactivity of ${\mathrm{SO}}_{4}{}^{2-}$, added to the medium in the form of $H_{2}{\mathrm{SO}}_{4}$. The molar rate of each of the reactions (1) to (34) can be expressed as shown in Equation (2).

(2) $$r_{i}=k_{i}\overset{K}{\underset{j=1}{\prod }}{\left[X_{j}\right]}^{{\vartheta^{\prime }}_{ji}}$$

$k_{i}$ is the kinetic constant related to the *i*th reaction from (1) to (34) and determined at the operating temperature, i.e., 25 °C, as indicated in Table 1.

Consequently, the molar rate of Fe(III) is expressed as shown in Equation (3).

(3) $$\frac{d\left[\mathrm{FeIII}\right]}{dt}=\frac{d\left[{\mathrm{Fe}}^{3+}\right]}{dt}+\frac{d\left[{\mathrm{FeOH}}^{2+}\right]}{dt}+\frac{d\left[\mathrm{Fe}\left(\mathrm{OH}\right){\mathrm{HO}}_{2}{}^{+}\right]}{dt}+\frac{d\left[\mathrm{Fe}{\left({\mathrm{HO}}_{2}\right)}^{2+}\right]}{dt}\;=\underset{i=1,\;4,\;8,\;9,\;10,\;11,\;12,\;28,\;34\;}{\sum }({v^{\prime \prime }}_{ki}-{v^{\prime }}_{ki})\;k_{i}\overset{K}{\underset{j=1}{\prod }}{\left[X_{j}\right]}^{{\vartheta^{\prime }}_{ji}}$$

The chemical mechanism presented in Table 1 expects the emergence of four radical species in the electrolyte, namely ${\mathrm{HO}}^{•}$, ${\mathrm{HO}}_{2}{}^{•}$, $O_{2}{}^{•-}$ and ${\mathrm{SO}}_{4}{}^{•-}$. In the absence of NBB, their molar yields are governed by the Equations (4)–(7), respectively.

(4) $$\frac{d\left[{\mathrm{HO}}^{•}\right]}{dt}=\underset{i=1,5,8,13,16,17,19,20,22,23,24,25\;}{\sum }({v^{\prime \prime }}_{ki}-{v^{\prime }}_{ki})\;k_{i}\overset{K}{\underset{j=1}{\prod }}{\left[X_{j}\right]}^{{\vartheta^{\prime }}_{ji}}$$

(5) $$\frac{d\left[{\mathrm{HO}}_{2}{}^{•}\right]}{dt}=\underset{i=4,5,7,9,10,14,15,16,18,19,26,27\;}{\sum }({v^{\prime \prime }}_{ki}-{v^{\prime }}_{ki})\;k_{i}\overset{K}{\underset{j=1}{\prod }}{\left[X_{j}\right]}^{{\vartheta^{\prime }}_{ji}}$$

(6) $$\frac{d\left[O_{2}{}^{•-}\right]}{dt}=\underset{i=6,7,11,12,15,17,18,20\;}{\sum }({v^{\prime \prime }}_{ki}-{v^{\prime }}_{ki})\;k_{i}\overset{K}{\underset{j=1}{\prod }}{\left[X_{j}\right]}^{{\vartheta^{\prime }}_{ji}}$$

(7) $$\frac{d\left[{\mathrm{SO}}_{4}{}^{•-}\right]}{dt}=\underset{i=22,23,24,25,26,27,28\;}{\sum }({v^{\prime \prime }}_{ki}-{v^{\prime }}_{ki})\;k_{i}\overset{K}{\underset{j=1}{\prod }}{\left[X_{j}\right]}^{{\vartheta^{\prime }}_{ji}}$$

In the presence of the dye in the electrolyte, Özcan and Özcan [20] proposed a chemical mechanism of chain reactions presented in Table 2, and whose intermediates were identified using the chromatographic technique.

The initiated by the oxidative attack of NBB by ${\mathrm{HO}}^{•}$ occurs at a rate constant k estimated by the same authors to $3.35\times 10^{7}\;{\mathrm{mol}}^{-1}\cdot m^{3}\cdot s^{-1}$.

Hence, the chemical kinetics related to ${\mathrm{HO}}^{•}$ in the presence of NBB and accounting for the initiation reaction of NBB degradation is expressed by Equation (8).

(8) $$\frac{d\left[{\mathrm{HO}}^{•}\right]}{dt}=\underset{i=1,5,8,13,16,17,19,20,22,23,24,25\;}{\sum }({v^{\prime \prime }}_{ki}-{v^{\prime }}_{ki})\;k_{i}\overset{K}{\underset{j=1}{\prod }}{\left[X_{j}\right]}^{{\vartheta^{\prime }}_{ji}}-k\left[{\mathrm{HO}}^{•}\right]\left[\mathrm{NBB}\right]$$

The formed systems of first-order differential equations are resolved using the 4th order Runge-Kutta algorithm considering the initial concentrations of $H_{2}O_{2}$, $H^{+}$, ${\mathrm{SO}}_{4}{}^{2-}$ and their equilibrium product ${\mathrm{HSO}}_{4}{}^{-}$ as indicated in Equation (9) [39].

(9) $${\left[{\mathrm{HSO}}_{4}{}^{-}\right]}_{0}=3.47\times 10^{1}\;M^{-1}\;{\left[H^{+}\right]}_{0}{\left[{\mathrm{SO}}_{4}{}^{2-}\right]}_{0}$$

## 4. Conclusions

In the present study, the Galvano-Fenton process was investigated from a mechanistic and parametric point of view by associating the experimental results of the degradation of NBB and the kinetics of Fe(III) to numerical modeling based on 37 reactions evolving at the electrodes and in the electrolyte. The GF total degradation of 5 mg·L^−1^ of NBB was observable during the experiment’s duration and explained by the spontaneous galvanic corrosion of iron plate sheets and the addition of hydrogen peroxide at 16.2, 32.4, 64.8, 97.2, and 162 µM. However, with a lower initial concentration of $H_{2}O_{2}$ of 3.24, 6.48, and 12.96 µM, total degradation was not achieved due to the rapid consumption of the Fenton reagent without providing the necessary yield of ${\mathrm{HO}}^{•}$ for the complete oxidation of the dye. The examination of the generation of hydroxyl radical revealed a maximum concentration attained within the first five minutes, with a gradual increase with the augmentation of the initial concentration of $H_{2}O_{2}$. The study of the effect of the initial concentration of NBB in the range of 5, 10, 15, and 30 mg·L^−1^ demonstrated that the increase of the concentration of the dye induces the extension of the necessary time for total degradation. However, the initial degradation rate showed an increasing trend until a concentration of 15 mg·L^−1^, it then decreased when passing to 30 mg·L^−1^ of NBB. This was explained by the diminution of the concentration of ${\mathrm{HO}}^{•}$, due at its turn to the simultaneous rapid consumptions of $H_{2}O_{2}$ and generated ${\mathrm{HO}}^{•}$. With high concentrations of NBB, it was concluded that a higher concentration of $H_{2}O_{2}$ is necessary to maintain the initial degradation rate proportional to the concentration of the dye. Finally, the experimental and simulated initial rates are correlated to more than 98%, which proves that the kinetic constant determined by Özcan and Özcan [20] and adopted in the present work describes well the initiation reaction of the oxidative attack of NBB by ${\mathrm{HO}}^{•}$.

## Figures and Tables

**Table 1:** Chemical schema of elementary reactions occurring in Galvano-Fenton process [36,37,38].

	Reactions	$k_{i}$	$\mathrm{Unit}\;\mathrm{of}\;k_{i}$
I	$\mathrm{Fe}\to {\mathrm{Fe}}^{2+}+2e^{-}$	Anodic reaction	
II	$2{\;H}^{+}+2e^{-}\to H_{2}$	Cathodic reaction	
1	${\mathrm{Fe}}^{2+}+H_{2}O_{2}\to {\mathrm{Fe}}^{3+}+{\mathrm{OH}}^{-}+{\mathrm{HO}}^{•}$	$6.3\times 10^{-2}$	${\mathrm{mol}}^{-1}\cdot m^{3}\cdot s^{-1}$
2	${\mathrm{Fe}}^{3+}+H_{2}O_{2}\to \mathrm{Fe}{\left({\mathrm{HO}}_{2}\right)}^{2+}+H^{+}$	$3.1\times 10^{4}$	${\mathrm{mol}}^{-1}\cdot m^{3}\cdot s^{-1}$
3	$\mathrm{Fe}{\left({\mathrm{HO}}_{2}\right)}^{2+}+H^{+}\to {\mathrm{Fe}}^{3+}+H_{2}O_{2}$	$1.0\times 10^{7}$	${\mathrm{mol}}^{-1}\cdot m^{3}\cdot s^{-1}$
4	$\mathrm{Fe}{\left({\mathrm{HO}}_{2}\right)}^{2+}\to {\mathrm{Fe}}^{2+}+{\mathrm{HO}}_{2}{}^{•}$	$2.3\times 10^{-3}$	$s^{-1}$
5	$H_{2}O_{2}+{\mathrm{HO}}^{•}\to {\mathrm{HO}}_{2}{}^{•}+H_{2}O$	$3.3\times 10^{4}$	${\mathrm{mol}}^{-1}\cdot m^{3}\cdot s^{-1}$
6	${\mathrm{HO}}_{2}{}^{•}\to O_{2}{}^{•-}+H^{+}$	$1.58\times 10^{5}$	$s^{-1}$
7	$O_{2}{}^{•-}+H^{+}\to {\mathrm{HO}}_{2}{}^{•}$	$1.0\times 10^{7}$	${\mathrm{mol}}^{-1}\cdot m^{3}\cdot s^{-1}$
8	${\mathrm{Fe}}^{2+}+{\mathrm{HO}}^{•}\to {\mathrm{Fe}}^{3+}+{\mathrm{OH}}^{-}$	$3.2\times 10^{5}$	${\mathrm{mol}}^{-1}\cdot m^{3}\cdot s^{-1}$
9	${\mathrm{HO}}_{2}{}^{•}+{\mathrm{Fe}}^{2+}+H_{2}O\to {\mathrm{Fe}}^{3+}+H_{2}O_{2}+{\mathrm{OH}}^{-}$	$1.2\times 10^{3}$	${\mathrm{mol}}^{-1}\cdot m^{3}\cdot s^{-1}$
10	${\mathrm{HO}}_{2}{}^{•}+{\mathrm{Fe}}^{3+}\to {\mathrm{Fe}}^{2+}+H^{+}+O_{2}$	$3.6\times 10^{2}$	${\mathrm{mol}}^{-1}\cdot m^{3}\cdot s^{-1}$
11	$O_{2}{}^{•-}+{\mathrm{Fe}}^{2+}+2H_{2}O\to {\mathrm{Fe}}^{3+}+H_{2}O_{2}+2{\;\mathrm{OH}}^{-}$	$1.0\times 10^{4}$	${\mathrm{mol}}^{-1}\cdot m^{3}\cdot s^{-1}$
12	$O_{2}{}^{•-}+{\mathrm{Fe}}^{3+}\to {\mathrm{Fe}}^{2+}+O_{2}$	$5.0\times 10^{4}$	${\mathrm{mol}}^{-1}\cdot m^{3}\cdot s^{-1}$
13	${\mathrm{HO}}^{•}+{\mathrm{HO}}^{•}\to H_{2}O_{2}$	$5.2\times 10^{6}$	${\mathrm{mol}}^{-1}\cdot m^{3}\cdot s^{-1}$
14	${\mathrm{HO}}_{2}{}^{•}+{\mathrm{HO}}_{2}{}^{•}\to H_{2}O_{2}+O_{2}$	$8.3\times 10^{2}$	${\mathrm{mol}}^{-1}\cdot m^{3}\cdot s^{-1}$
15	$O_{2}{}^{•-}+H^{+}\to {\mathrm{HO}}_{2}{}^{•}$	$1.0\times 10^{7}$	${\mathrm{mol}}^{-1}\cdot m^{3}\cdot s^{-1}$
16	${\mathrm{HO}}^{•}+{\mathrm{HO}}_{2}{}^{•}\to O_{2}+H_{2}O$	$7.1\times 10^{6}$	${\mathrm{mol}}^{-1}\cdot m^{3}\cdot s^{-1}$
17	${\mathrm{HO}}^{•}+O_{2}{}^{•-}\to O_{2}+{\mathrm{OH}}^{-}$	$1.01\times 10^{7}$	${\mathrm{mol}}^{-1}\cdot m^{3}\cdot s^{-1}$
18	${\mathrm{HO}}_{2}{}^{•}+O_{2}{}^{•-}+H_{2}O\to H_{2}O_{2}+O_{2}+{\mathrm{OH}}^{-}$	$9.7\times 10^{4}$	${\mathrm{mol}}^{-1}\cdot m^{3}\cdot s^{-1}$
19	${\mathrm{HO}}_{2}{}^{•}+H_{2}O_{2}\to O_{2}+{\mathrm{HO}}^{•}+H_{2}O$	$5.0\times 10^{-4}$	${\mathrm{mol}}^{-1}\cdot m^{3}\cdot s^{-1}$
20	$O_{2}{}^{•-}+H_{2}O_{2}\to O_{2}+{\mathrm{HO}}^{•}+{\mathrm{OH}}^{-}$	$1.3\times 10^{-4}$	${\mathrm{mol}}^{-1}\cdot m^{3}\cdot s^{-1}$
21	${\mathrm{Fe}}^{2+}+{\mathrm{SO}}_{4}{}^{2-}\to {\mathrm{FeSO}}_{4}$	$2.29\times 10^{8}$	${\mathrm{mol}}^{-1}\cdot m^{3}\cdot s^{-1}$
22	${\mathrm{SO}}_{4}{}^{2-}+{\mathrm{HO}}^{•}\to {\mathrm{SO}}_{4}{}^{•-}+{\mathrm{OH}}^{-}$	$1.4\times 10^{4}$	${\mathrm{mol}}^{-1}\cdot m^{3}\cdot s^{-1}$
23	${\mathrm{HSO}}_{4}{}^{-}+{\mathrm{HO}}^{•}\to {\mathrm{SO}}_{4}{}^{•-}+H_{2}O$	$3.5\times 10^{2}$	${\mathrm{mol}}^{-1}\cdot m^{3}\cdot s^{-1}$
24	${\mathrm{SO}}_{4}{}^{•-}+H_{2}O\to H^{+}+{\mathrm{SO}}_{4}{}^{2-}+{\mathrm{HO}}^{•}$	$3.0\times 10^{5}$	$s^{-1}$
25	${\mathrm{SO}}_{4}{}^{•-}+{\mathrm{OH}}^{-}\to {\mathrm{SO}}_{4}{}^{2-}+{\mathrm{HO}}^{•}$	$1.4\times 10^{4}$	${\mathrm{mol}}^{-1}\cdot m^{3}\cdot s^{-1}$
26	${\mathrm{SO}}_{4}{}^{•-}+H_{2}O_{2}\to {\mathrm{SO}}_{4}{}^{2-}+H^{+}+{\mathrm{HO}}_{2}{}^{•}$	$1.2\times 10^{4}$	${\mathrm{mol}}^{-1}\cdot m^{3}\cdot s^{-1}$
27	${\mathrm{SO}}_{4}{}^{•-}+{\mathrm{HO}}_{2}{}^{•}\to {\mathrm{SO}}_{4}{}^{2-}+H^{+}+O_{2}$	$3.5\times 10^{6}$	${\mathrm{mol}}^{-1}\cdot m^{3}\cdot s^{-1}$
28	${\mathrm{SO}}_{4}{}^{•-}+{\mathrm{Fe}}^{2+}\to {\mathrm{Fe}}^{3+}+{\mathrm{SO}}_{4}{}^{2-}$	$3.0\times 10^{5}$	${\mathrm{mol}}^{-1}\cdot m^{3}\cdot s^{-1}$
29	${\mathrm{FeSO}}_{4}\to {\mathrm{Fe}}^{2+}+{\mathrm{SO}}_{4}{}^{2-}$	$1.0\times 10^{10}$	$s^{-1}$
30	${\mathrm{Fe}}^{3+}+H_{2}O\to {\mathrm{FeOH}}^{2+}+H^{+}$	$2.9\times 10^{7}$	$s^{-1}$
31	${\mathrm{FeOH}}^{2+}+H^{+}\to {\mathrm{Fe}}^{3+}+H_{2}O$	$1.0\times 10^{7}$	${\mathrm{mol}}^{-1}\cdot m^{3}\cdot s^{-1}$
32	${\mathrm{FeOH}}^{2+}+H_{2}O_{2}\to \mathrm{Fe}\left(\mathrm{OH}\right){\mathrm{HO}}_{2}{}^{+}+H^{+}$	$2.0\times 10^{3}$	${\mathrm{mol}}^{-1}\cdot m^{3}\cdot s^{-1}$
33	$\mathrm{Fe}\left(\mathrm{OH}\right){\mathrm{HO}}_{2}{}^{+}+H^{+}\to {\mathrm{FeOH}}^{2+}+H_{2}O_{2}$	$1.0\times 10^{7}$	${\mathrm{mol}}^{-1}\cdot m^{3}\cdot s^{-1}$
34	$\mathrm{Fe}\left(\mathrm{OH}\right){\mathrm{HO}}_{2}{}^{+}\to {\mathrm{Fe}}^{2+}+{\mathrm{HO}}_{2}{}^{•}+{\mathrm{OH}}^{-}$	$2.3\times 10^{-3}$	$s^{-1}$

**Table 2:** Detailed chain mechanism of oxidation of naphthol blue black initiated by hydroxyl radical attack [20].

NBB+ HO^•^ → aniline + 4-nitroaniline + multi-substituted naphthalene ring	**Initiation**
Aniline → phenol	**Propagation**
Aniline → *p*-hydroquinone	
Phenol → *p*-hydroquinone	
*p*-hydroquinone → *p*-benzoquinone	
Phenol → catechol	
Phenol → resorcinol	
Catechol → 1, 2, 3 benzenetriol	
Catechol → 1, 2, 4 benzenetriol	
Resorcinol→ 1, 2, 3 benzenetriol	
Resorcinol→ 1, 2, 4 benzenetriol	
*p*-hydroquinone → maleic acid, butanedioic acid, butenedioic acid, glycolic acid, malic acid, glyceric acid and 3-hydroxypropanoic acid.	
*p*-benzoquinone → maleic acid, butanedioic acid, butenedioic acid, glycolic acid, malic acid, glyceric acid and 3-hydroxypropanoic acid.	
1, 2, 3 benzenetriol → maleic acid, butanedioic acid, butenedioic acid, glycolic acid, malic acid, glyceric acid and 3-hydroxypropanoic acid.	
1, 2, 4 benzenetriol → maleic acid, butanedioic acid, butenedioic acid, glycolic acid, malic acid, glyceric acid and 3-hydroxypropanoic acid.	
maleic acid, butanedioic acid, butenedioic acid, glycolic acid, malic acid, glyceric acid and 3-hydroxypropanoic acid → oxalic acid, oxamic acid	
oxalic acid, oxamic acid → carbon dioxide, water, ammonium, nitrate, sulfate	**Termination**

## Data Availability

Not applicable.
